# A Whole Genome DArTseq and SNP Analysis for Genetic Diversity Assessment in Durum Wheat from Central Fertile Crescent

**DOI:** 10.1371/journal.pone.0167821

**Published:** 2017-01-18

**Authors:** Faheem Shehzad Baloch, Ahmad Alsaleh, Muhammad Qasim Shahid, Vahdettin Çiftçi, Luis E. Sáenz de Miera, Muhammad Aasim, Muhammad Azhar Nadeem, Husnu Aktaş, Hakan Özkan, Rüştü Hatipoğlu

**Affiliations:** 1 Department of Field Crops, Faculty of Agricultural and Natural Science, Abant İzzet Baysal University, Bolu, Turkey; 2 Department of Biotechnology, Faculty of Agriculture, University of Çukurova, Adana, Turkey; 3 State Key Laboratory for Conservation and Utilization of Subtropical Agro-bioresources, South China Agricultural University, Guangzhou, China; 4 Department of Molecular Biology, Faculty of Biology, University of León, León, Spain; 5 Department of Biotechnology, Faculty Science, Necmettin Erbakan University, Konya, Turkey; 6 Artuklu University Vocational Higher School of Kızıltepe, Mardin, Turkey; 7 Department of Field Crops, Faculty of Agriculture, University of Çukurova, Adana, Turkey; USDA-ARS Southern Regional Research Center, UNITED STATES

## Abstract

Until now, little attention has been paid to the geographic distribution and evaluation of genetic diversity of durum wheat from the Central Fertile Crescent (modern-day Turkey and Syria). Turkey and Syria are considered as primary centers of wheat diversity, and thousands of locally adapted wheat landraces are still present in the farmers’ small fields. We planned this study to evaluate the genetic diversity of durum wheat landraces from the Central Fertile Crescent by genotyping based on DArTseq and SNP analysis. A total of 39,568 DArTseq and 20,661 SNP markers were used to characterize the genetic characteristic of 91 durum wheat land races. Clustering based on Neighbor joining analysis, principal coordinate as well as Bayesian model implemented in structure, clearly showed that the grouping pattern is not associated with the geographical distribution of the durum wheat due to the mixing of the Turkish and Syrian landraces. Significant correlation between DArTseq and SNP markers was observed in the Mantel test. However, we detected a non-significant relationship between geographical coordinates and DArTseq (r = -0.085) and SNP (r = -0.039) loci. These results showed that unconscious farmer selection and lack of the commercial varieties might have resulted in the exchange of genetic material and this was apparent in the genetic structure of durum wheat in Turkey and Syria. The genomic characterization presented here is an essential step towards a future exploitation of the available durum wheat genetic resources in genomic and breeding programs. The results of this study have also depicted a clear insight about the genetic diversity of wheat accessions from the Central Fertile Crescent.

## Introduction

Cultivated wheats and their close wild relatives belong to the genus *Triticum* L., a member of the tribe Triticeae, which contains about 300 species [1]. Durum wheat (2n = 4X = 28, AABB), the most common cultivated form of allotetraploid wheat, consumed as macaroni and semolina products, has constituted the ‘*founder crop assemblage*’ on which ‘Old World’ agriculture was built [2,3]. Durum wheat, which is a free threshing wheat, arose in the eastern Mediterranean [4] and replaced its ancestor *T*. *dicoccum* to take place as the major cultivated form of allotetraploid wheat based on the studies of restriction fragment length polymorphism data (RFLP) [2]. Southeastern part of Turkey and Northern Syria are the crucial regions with respect to wheat domestication [5].

Geographical expansion of durum wheat was intimately associated with human migrations. It is cultivated mainly in the marginal areas of Mediterranean region, Southern Europe, and North Africa, while more recently it has started to expand to Southern Asia. It played a critical role in the food of local people of Mediterranean Basin, where about 75% of the world’s durum wheat is produced [6]. Here, durum wheat is extensively used both for classical farming practices and in the diet of local population.

The genetic diversity analysis of plants is a critical component of plant genetics, breeding, conservation and evolution [7]. However, as in other crops, genetic diversity of wheat has declined following domestication and intense selection in modern plant breeding programs. This has caused fall in the number of genetically distinct, locally well-adapted landraces and decrease on-farm genetic variability [8, 9]. In the previous decades, an enormous number of durum wheat cultivars has been created by natural selection, mainly established on huge yield, pathogen resistance and technological qualities. However, reliance of breeding programs on a small number of elite cultivars has eroded the genetic base of crops throughout the world. For an example, Autrique and his colleagues [10] have studied 51 cultivars retrieved from the CIMMYT/ICARDA breeding program and found that the same 15 ancestors were present in the pedigree of at least 80% of the cultivars, with five being found in all of them. The genetic diversity and molecular characterization of durum wheat landraces have been done in Turkey and Syria in earlier studies by using different molecular markers such as SSR, AFLP, RAPD, ISSR [11–13]. The results have depicted that durum wheat in Turkey and Syria harbor high diversity, which is not surprising, as South Eastern Turkey and Northern part of Syria are core areas of wheat domestication and diversity. Nonetheless, the landraces assessed so far are only as a limited subset of accessible assets; additionally, they come from small geographic regions and do not allow for the studies of the genetic structure of durum wheat landraces from Central Fertile Crescent (Turkey and Syria).

More recently, a novel approach named genotyping-by-sequencing (GBS) has been developed to perform simultaneous SNP discovery and genotyping [14]. The GBS approach is a united one-step procedure of SNP discovery and genotyping and constitutes a rapid, high-throughput and cost-effective tool for a genome-wide analysis of genetic diversity, especially for non-model species and germplasm sets. These features of GBS are encouraging and advantageous for genetic diversity studies in plants with informative sets of markers [7]. Many processes have been advanced to cut down genome complexity; nonetheless, the DArTseq method has brought a momentous influence via intelligent selection of the genome fraction corresponding predominantly to active genes. Classic DArT markers have been substituted by DArTseq markers based on genotyping by sequencing. DArTseq and SNP markers based on GBS technology have been successfully applied for linkage mapping, QTL identification in biparental mapping population, genome wide association studies (GWAS), genetic diversity studies in wheat [15–18] and many other crops [19,20] and as well marker-assisted and genomic selection. However, genetic diversity and population structure of the durum wheat landraces from Central Fertile Crescent revealed by DArTseq and SNP markers together have not been reported yet.

The Fertile Crescent, particularly the Eastern Mediterranean area, is considered to be the fundamental center of wheat domestication and diversity [6]. Albeit the emphasis of the genetic diversity from this region, there is a noticeable scarcity of information on the genetic structure of the durum wheat gene pool from this locality. A few studies have described the genetic diversity of Turkish and Syrian durum wheat landraces in terms of allele number and specific alleles. It is commonly reported that there is a trend of reduction of genetic diversity in self-pollinated crops, such as durum wheat, due to the selection pressure for the economically important traits. Therefore, this study has aimed to evaluate the genetic diversity of a collection of durum wheat landraces from Central Fertile Crescent (Turkey and Syria) using two types of molecular markers, SNP and DArTseq.

## Material and Methods

### Plant material

A diversity panel was assembled comprising of DNAs of durum wheat landraces representing the various geographical regions of Turkey and Syria (Central Fertile Crescent). The durum wheat landraces originated from a wide range of ecological conditions of soil, temperature and water availability, representing the natural distribution of durum wheat from Syria and Turkey. 91 durum wheat landraces including 32 landraces collected from various geographical areas of Syria and 59 landraces belonging to different climatic zones of Turkey were used as plant material. The collected set of durum wheat landraces used in this study represents the samples collected by the ICARDA plant genetic resources team, which are preserved at ICARDA gene bank, Aleppo, Syria. All the data about the origin, collection site, and passport information are shown in Table 1. All durum wheat landraces were grown at the research and implementation area of Abant İzzet Baysal University, Bolu during the growing season of 2015, in order to multiply seeds for further studies. Single plants were selected from each durum wheat landrace and used for the diversity analysis. The seeds are available upon request from the corresponding author.

**Table 1:** Origin, collection sites and geographical coordinates of durum wheat landraces from Central Fertile Crescent used in this study.

No	Gene bank Number	Origin	Province	Collection site	Longitude	Latitude	Altitude
T01	82715	Turkey	Nevşehir	Avanos	E3451	N3845	
T02	82738	Turkey	Aksaray	Aksaray	E3402	N3823	
T03	82880	Turkey	Gumuşhane	Bayburt	E04015	E4016	
T04	83017	Turkey	Eskişehir	Eskişehir	E3116	N39 38	
T05	83033	Turkey	Konya	Kulukkoy	E033 05	N3906	
T06	84111	Turkey	Niğde	Niğde	E3441	N37 59	
T07	84263	Turkey	Manisa	Manisa	E2725	N3837	45
T08	84273	Turkey	Uşak	Uşak	E2925	N38 42	
T09	84510	Turkey	Mardin	Derbesiye (Şenyurt)	E4039	N37 06	
T10	84733	Turkey	Tekirdağ	Bekirli	E28 05	N4114	
T11	84745	Turkey	Bursa	Karacabey	E2822	N4013	
T12	85621	Turkey	K.Maraş	Yokoul-Yemez market	E0300	N3800	
T13	85705	Turkey	Malatya	44 km from Maras	E036 47	N3715	850
T14	85706	Turkey	Elaziğ	15 km E of Elazig	E3915	N38 41	1000
T15	86062	Turkey	Erzurum		E4117	N39 54	
T16	86427	Turkey	Trabzon	Komera	E039 50	N4055	
T17	87552	Turkey	Konya	Ereğli	E34 03	N3730	
T18	87758	Turkey	Sinop	mountainside, 75 km S of Sinop	E3503	N41 28	732
T19	88269	Turkey	Ankara	5 km W of Sorgun-Ankara	E32 14	N4020	1050
T20	88271	Turkey	Yozgat	78 km W of Yozgat	E34 08	N3949	650
T21	88276	Turkey	Adana	35 km NE of Osmaniye	E3626	N3710	850
T22	89198	Turkey	İzmir	Bornıova	E27 13	N3828	25
T23	89223	Turkey	Konya	Konya	E3228	N3752	
T24	89272	Turkey	Bilecik	Soğut	E3010	N40 02	
T25	89955	Turkey	Siirt		E41 57	N3756	
T26	92494	Turkey	Ankara	Nallihan	E31 22	N4012	
T27	92496	Turkey	Kirikkale	Çerikli	E3400	N39 54	
T28	95959	Turkey	Adıyaman	3 km NE Nariance village (Nimrud road)	E03844	N3747	525
T29	95961	Turkey	Denizli	13 km N Buldan junction	E0285248	N380239	385
T30	95963	Turkey	Diyarbakir	8 km SW of Dicle	E0400345	N382215	950
T31	95978	Turkey	Antalaya	Agullu village; 8 km NE of Kas	E2941	N3614	500
T32	95994	Turkey	K.Maraş	16 km NE Pazarcik	E3724	N3731	800
T33	96006	Turkey	Urfa	3 km SW Hilvan	E0391219	N365137	600
T34	96009	Turkey	Antakya	8 km SE Iskenderun	E03611 24	N3631 33	350
T35	96045	Turkey	Manisa	1 km E of road junction to Selendi on Izmir highway	E2852	N3838	490
T36	96047	Turkey	Cankiri	14 km N Llgaz	E03341 41	N4032 32	1120
T37	96052	Turkey	Samsun	14 km N Sinop/Corum border	E3456	N4117	350
T38	96059	Turkey	Kars	11 km NW junction at Kagizman	E04300 57	N4010 45	1800
T39	96061	Turkey	Amasya	6 km S Boyabat; Samsun-Boyabat junction	E03537 46	N4051 46	400
T40	96073	Turkey	Zonguldak	7 km W Devrek	E03154 55	N41 14 14	500
T41	96074	Turkey	Canakkale	16 km SE Ayvacik	E02631 16	N3934 03	310
T42	96081	Turkey	Corum	Highway junction and sign to Karacaoglan village or 6 km S Corum-Sinop province border	E03451 33	N411231	300
T43	96093	Turkey	Bolu	10 km E Akcakoca	E03115 30	N4105 30	20
T44	96105	Turkey	Gaziantep	2 km N Gaziantep towards Yavuzeli	E03721 09	N3706 05	940
T45	96122	Turkey	Diyarbakir	19 km SE of Diyarbakir-Bismil junction on road to Bismil	E04024 22	N3739 03	825
T46	96126	Turkey	Kirsehir	12 km NW of Kirsehir	E3410	N3914	1000
T47	97411	Turkey	Edirne	1 km S Sarayakpinar	E0262753	N414616	190
T48	97447	Turkey	Kayseri	Pinarbasi	E3624	N3843	
T49	97451	Turkey	Urfa	Suruc	E3824	N3659	
T50	97456	Turkey	Icel	Icel	E3438	N3648	
T51	99032	Turkey	Konya	27 km NE Beysehir	E3149	N3735	1250
T52	127613	Turkey	Ankara	village Beypazari	E 3155	N 4010	
T53	127634	Turkey	Tokat	village Bazar key	E 3645	N 40 31	894
T54	127648	Turkey	Sivas	village Sarac Kouy	E 3612	N 3920	1400
T55	42218	Turkey	Agri	24 km E of Malazgirt	E04244	N3908	1610
T56	84271	Turkey	Tekirdag	Malkara	E2654	N4054	
T57	44961	Turkey	Urfa	5 km N of Urfa	E3847	N3712	600
T58	45033	Turkey	Hakkari	2 km W of Yuksekova-Daglica junction	E4412	N3734	1850
T59	45090	Turkey	Van	2 km SE Van towards Gurpinar	E4322	N3830	1810
S60	84857	Syria	Damascus	Damascus	E3618	N3330	
S61	95789	Syria	Sweida	Ruddima	E363435	N33015	840
S62	95791	Syria	Sweida	Welgha	E363115	N324440	930
S63	95798	Syria	Dar’a	Orika	E3628	N32 23	850
S64	95800	Syria	Dar’a	Hamir	E361735	N325635	660
S65	95804	Syria	Dar’a	Danun; 5 km S	E361345	N331720	810
S66	95807	Syria	Damascus	Deir Makir	E360130	N331400	940
S67	95808	Syria	Damascus	Saas'a; 3 km W	E360015	N331815	980
S68	95810	Syria	AlQunaytirah	Trunje	E355100	N331355	1060
S69	95841	Syria	Dayr Az Zawr	Salu Regional Research Station	E402019	N350835	230
S70	95843	Syria	Dayr Az Zawr	Salu Regional Research Station	E402019	N350835	230
S71	95849	Syria	Al Hasakah	Tall Bedar village	E403456	N364412	420
S72	95852	Syria	Al Hasakah	Ghweitly	E4040	N36 55	460
S73	95882	Syria	Aleppo	Blass village	E370940	N360020	440
S74	95885	Syria	Idlib	Sarmada	E364248	N361127	440
S75	95886	Syria	Idlib	Barisha	E363800	N361100	610
S76	95891	Syria	Aleppo	Bianon	E370225	N362040	410
S77	95895	Syria	Aleppo	Katmeh	E365715	N363525	600
S78	95916	Syria	Homs	Kafr Nan	E363830	N345315	450
S79	95917	Syria	Homs	Tall Douw	E363130	N345240	410
S80	95925	Syria	Hama	Jnan	E365010	N350445	350
S81	95929	Syria	Hama	Kanafez	E371600	N351315	520
S82	95947	Syria	Tartous	Doir Al Mouliha	E361500	N345200	570
S83	95948	Syria	Tartous	Tall Altrmous	E360700	N344320	70
S84	96149	Syria	Raqqa	Mansura; 12 km S	E384430	N355035	330
S85	96150	Syria	Raqqa	Hamam	E384630	N355410	320
S86	96168	Syria	Lattakia	Bahlulieh	E355730	N353800	45
S87	98853	Syria	Al Qunaitra	Hineh village; 4 km N of Hiveh	E3557	N3321	1040
S88	98937	Syria	Lattakia	2 km before Ain El Wadi	E361030	N353630	1000
S89	110711	Syria	Al Hasakah	Markadeh village	E404555	N354401	280
S90	110715	Syria	Al Hasakah	Ayn al Bazuq; 4 km after Malkieh	E421037	N371234	460
S91	110706	Syria	Damascus	Kutaifa; 10 km after town	E0363412	N334648	1020

### DNA extraction

Genomic DNA was obtained from each single selected plant of each landrace according to CTAB protocol [21] with some modifications [18]. The quality and quantity of the extracted DNA were checked using DS-11 FX Series Spectrophotometer/Fluorometer (Denovix, Wilmington, DE, USA) and further confirmed on 0.8% agarose gel run in TAE buffer at 80 V. Short or degraded DNA was eliminated and DNA concentrations of 50 ng μl^-1^.

### Genotyping by sequencing (GBS) analysis

Genotyping by sequencing analysis of the 91 durum wheat landraces was performed by using a whole genome profiling service for SNP and DArTseq markers. 100 μl of 50 ng μl^-1^ was sent to Diversity Array Technology (http://www.diversityarrays.com/) for SNP and DArTseq analysis following the protocol as described by [15]. Raw sequence data of each clone is given in the S1 Table.

### Statistical analysis

All the images from DArTseq and SNP platforms were analyzed using DArTsoft v.7.4.7 (DArT P/L, Canberra, Australia). The DArTseq and SNP markers were scored using DArTsoft as binary data (1/0), indicating the presence or absence of a marker in the genomic representation of each sample as described by Akbari et al. [15]. The DArT software automatically has computed several quality parameters for each DArTseq and SNP marker, such as call rate, polymorphic information content (PIC), and reproducibility of both markers.

DArTseq and SNP markers obtained for each landrace were used to calculate the genetic diversity using the statistical software ‘R’. Genetic distance among the durum wheat landraces was measured from the proportion of shared alleles obtained from DArTseq and SNP marker data set by using Jaccard similarity index and transformed into distance by using the vegdist function of vegan package in R. Correlation matrices between the both marker systems were determined by using the Mantel test as implemented in the ape package of statistical software program ‘R’ employing 10,000 random iterations in the non-parametric test calculator.

Pair-wise genetic distances among durum wheat landraces was used to construct the Neighbor Joining trees using the ape package implemented in R. The computer software MEGA 6.0 [22] was used to visualize and edit the resulting tree.

### Population structure

In order to have a clear picture about the genetic structure of durum wheat landraces collected, we applied the Bayesian model-based clustering algorithm implemented in the STRUCTURE software. Admixture and shared allele frequencies model was used to determine the number of clusters (K) in the range from 1 to 12. For each run, the initial burn-in period was set to 500 with 500,000 MCMC (Markov chain Monte Carlo) iterations, with no prior information on the origin of individuals. The ΔK method was used to determine the most suitable value of K as implemented in Structure Harvester [23].

Turkish and Syrian landraces were grouped in two populations (Turkey and Syria). Differentiation and significance levels were assessed by calculating the pair-wise F_ST_ values. Adonis function of the vegan package was used as an alternative to AMOVA (Analysis of molecular variance) to estimate the variance between populations of both regions.

## Results

### DArTseq diversity and population structure

**Fig 1:**
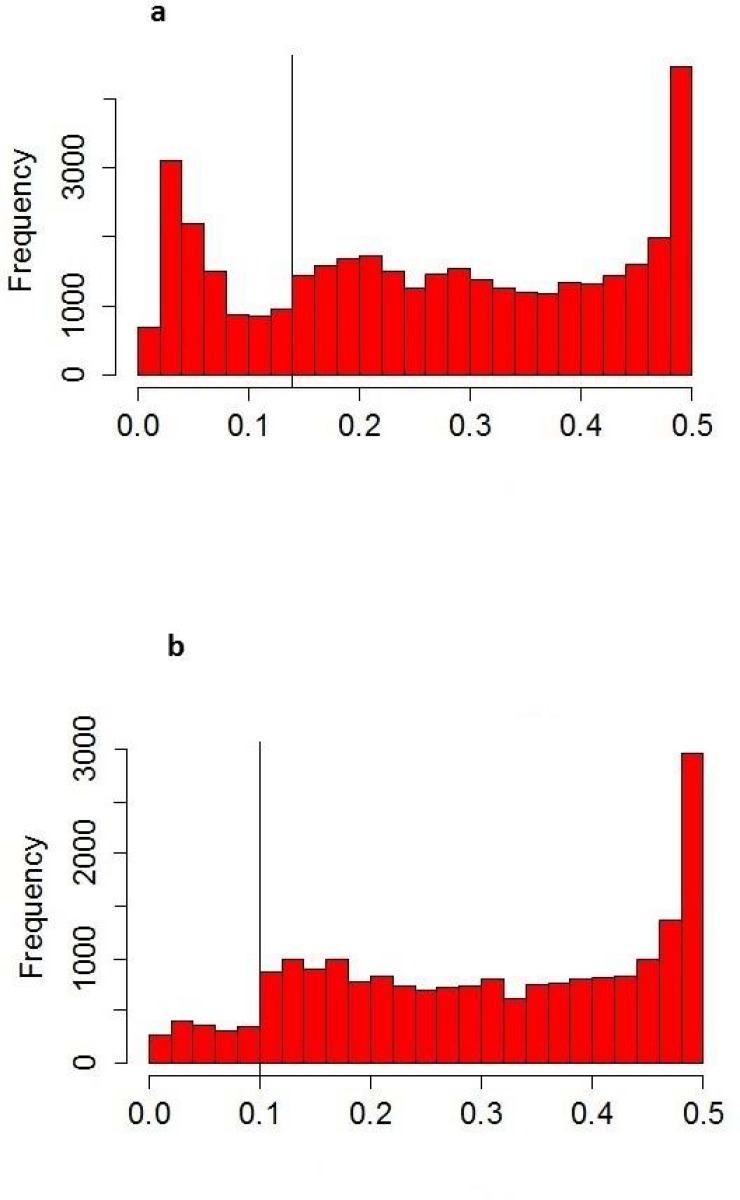
Frequency distribution of PIC values of (a) DArTseq and (b) SNP).

A total of 56,334 DArTseq markers were generated for the 91 landraces representing different ecological and geographical areas of Turkey and Syria. The chromosomal locations of some DArTseq markers (14,054) have been provided by the Diversity Array Technology, Pvt, Ltd, Australia. However, most of the DArTseq markers have been reported first time in these accessions and their chromosomal location have not been described yet. A total of DArT 39,568 markers showed polymorphism and were used for further analysis. The overall polymorphism information contents (PIC) of the DArTseq markers was 0.265 and the median 0.000. The distribution of DArTseq markers according to their PIC values is shown in Fig 1A. The quality of DArTseq markers was assessed by different quality parameters, such as call rate and reproducibility percentage. The average call rate of the all markers, which shows the loyalty of the final scores and produces the number of scored slides versus the maximum number of potential scores, was 0.95%, ranging from 0.766 to 1.0%. The reproducibility of all the DArTseq markers used in the analysis was 1, showing consistent marker score and a hundred percent reproducibility.

**Fig 2:**
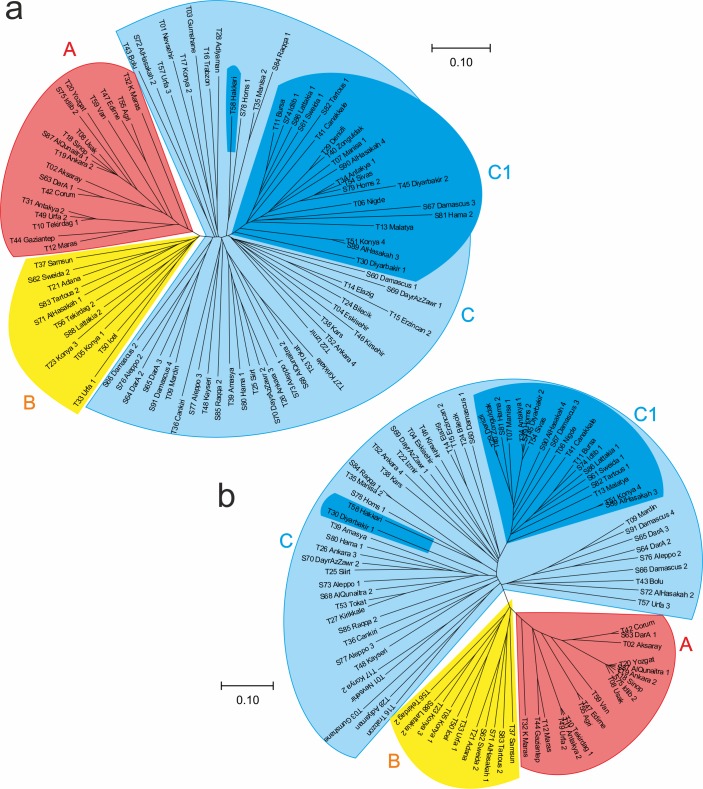
Neighbour joining analysis of 91 durum wheat landrces based (a) DArTseq (b) SNP markers.

The DArTseq marker data set was used to calculate the Jaccard genetic distance index values among the 91 durum landraces. The average genetic distance among all landraces was 0.494 and the highest genetic distance (0.687) was calculated between a Turkish landraces “Gumushane” and Syrian landrace ‘”Hama_2”, while the minimum genetic distance (0.0486) was found between the Turkish landrace “Konya_4” and a Syrian landrace “AlHasakah”. A dendrogram was constructed for all the landraces based on the Jaccard genetic distance. DArTseq based Neighbor joining analysis grouped all durum landraces into 3 clusters, A, B and C. Cluster A harbored 18 landraces (15 from Turkey and 3 from Syria), and cluster B consisted of 11 landraces with 7 from Turkey and 4 from Syria. Cluster C is a heterogeneous group with the rest of landraces (Fig 2A). There was no clear differentiation between Turkish and Syrian landraces. Moreover, grouping of these landraces was not according to their center of origin and geographical regions.

**Fig 3:**
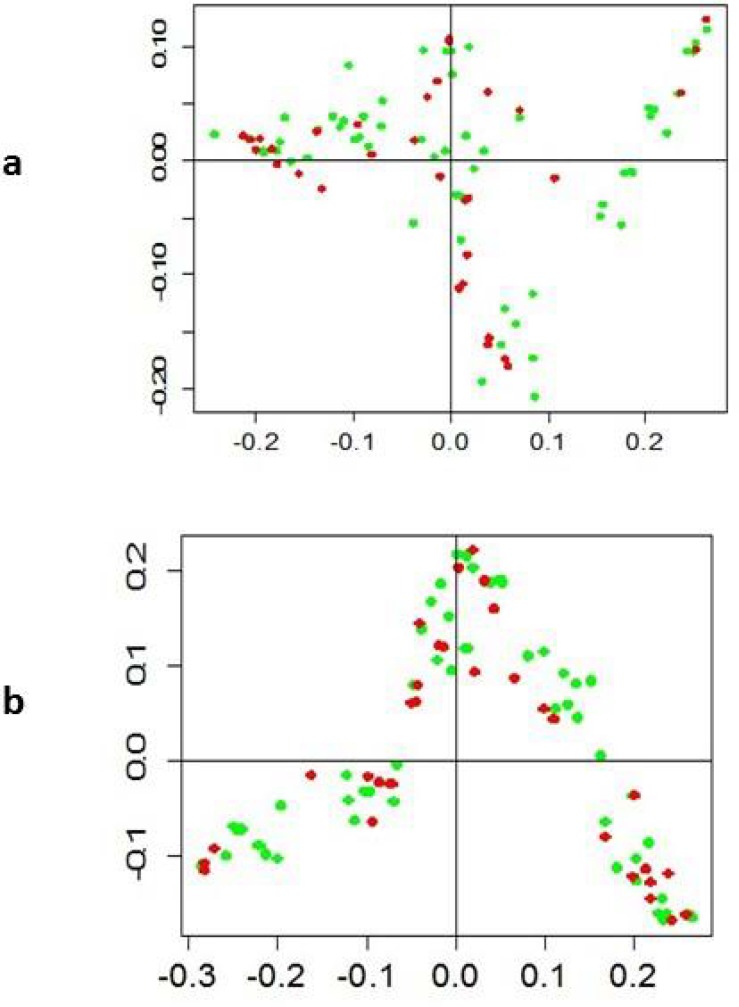
Principal coordinate analysis of durum wheat landraces based on (a) DArTseq (b) SNP markers.

After we got an idea about the diversity among the landraces of each country, we estimated the genetic distances among the pairs of landraces between each country and we found that both countries have the same level of genetic diversity and there was no clear differentiation among the landraces of both countries. The average genetic distance between Turkish and Syrian landraces was 0.494. Mean Jaccard genetic distance among Turkish landraces was 0.492 and it was slightly higher for Syrian landraces (0.496). When we talk about the genetic distance among pairs of landraces within each country, the genetic distance ranged from 0.065 to 0.658, whereas in the case of Syria, it varied from 0.049 to 0. 687. To have more insight into the landraces grouping and the pattern of variation, principal coordinate analysis (PCoA) was utilized to measure the variation in durum wheat collection based on the DArTseq markers. In our study, first two principal coordinates explained 56% of the total variations (Fig 3A). Using the first two axes, the durum wheat landraces from Syria and Turkey were mixed in the grouping. This is in agreement with the Neighbor Joining analysis, where the grouping of the Turkish and Syrian landraces was not accordingly to their geographical provenance and center of origin.

**Fig 4:**
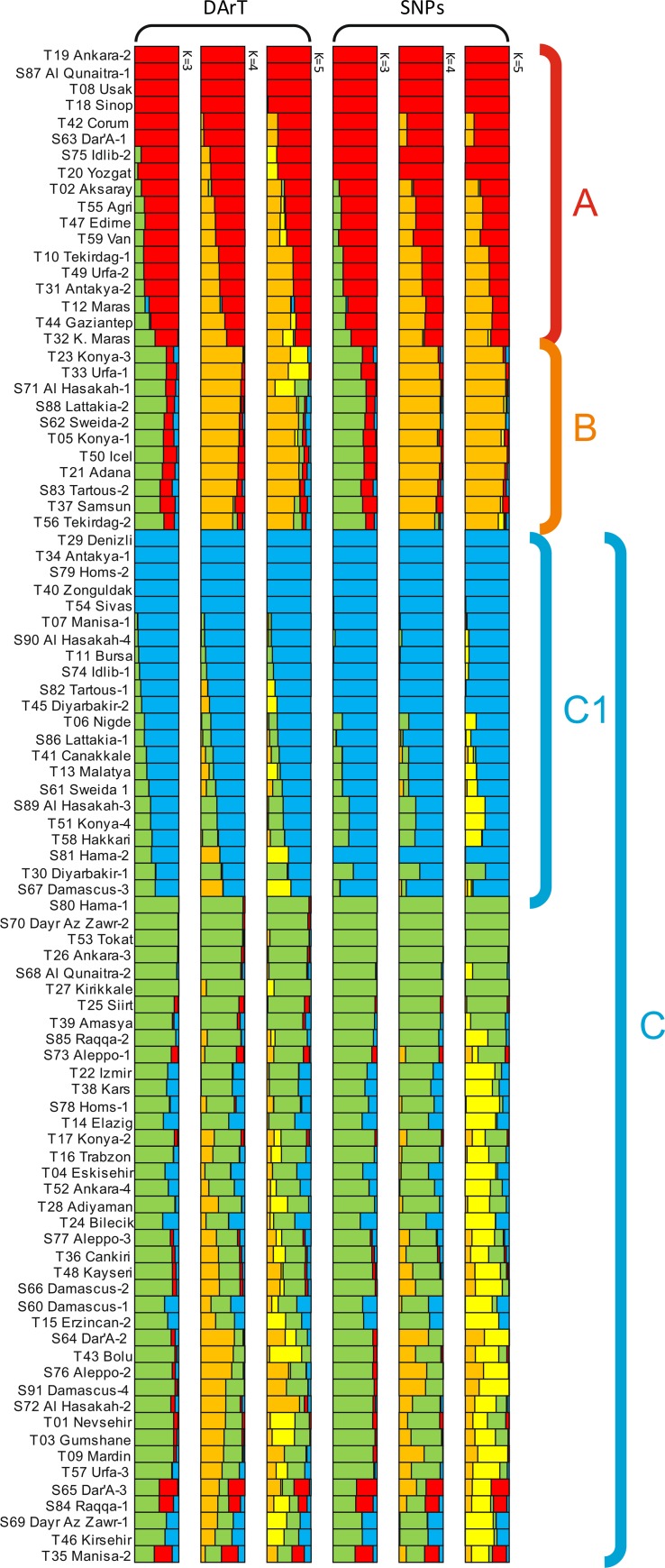
Structure analysis of durum wheat landraces based on DArTseq and SNP markers.

To have an idea about the population genetic structure of the Turkish and Syrian landraces, Bayesian clustering modeling was used as implemented in STRUCTURE using the DArTseq markers data set. The determination of suitable number of clusters in STRUCTURE analysis is bit tricky task, as there is no clear and repeatable value of K. In order to find the suitable value of K, the number of cluster (K) was plotted against ΔK according to Evanno et al. [23]. The highest value of ΔK was observed at K = 3, K = 4 and K = 5 for DArTseq markers. According to the value of K = 3, durum wheat collection was grouped into three subpopulations, while at K = 4 four and at K = 5 five subpopulations were observed. The ΔK value as described by [23] was the highest when assuming five clusters (ΔK(5) = 161.1). It was reduced with the number of clusters (K) to 4 ((ΔK(4) = 110) and two fold reduction occurred in ΔK value (ΔK(3) = 82) with 3 clusters. Most of the accessions were assigned to group three at any of the three K values (Fig 4).

### Genetic diversity and population structure based on SNP

A total of 20,661 SNP markers were generated by DArT sequencing and used for evaluation of genetic diversity of 91 durum wheat landraces representing the diversity present in Turkey and Syria. As with DArTseq markers, the SNP dataset used in this research was not previously used or mapped for wheat. 9,791 SNP markers were previously mapped and their chromosomal locations were provided by Diversity Array Technology, Pvt, Ltd, Australia. All of the SNP markers were polymorphic and were consistent. However, most of the SNPs (52.6%) reported here are new and their chromosomal locations are still unknown. Several quality parameters have been generated by the DArT software, including polymorphism information contents (PIC), call rate and reproducibility of each marker. PIC value of the SNP markers ranged from 0.011 to 0.5, with an average of 0.302. The frequency distribution of PIC values of the 20,661 SNP markers used in this study is shown in the histogram (Fig 1B).

Genetic distances (D) were calculated among all pairs of durum wheat landraces, based on the shared-allele distances. The highest genetic distance was detected between the Turkish landrace “Gumushane” and the Syrian landrace “Damascus_4”, with a value of 0.68, while the mean genetic distance was 0.51. We continued with the Neighbor Joining analysis based on the Jaccard genetic distance and all the landraces were grouped into three clusters A, B and C with 18, 11, and 62 landraces respectively (Fig 2B). Clustering of the durum wheat landraces was not in accordance with their provenances. Similarly, PCoA also showed consistent results and illustrated mixing of the landraces from both countries (Fig 3B).

SNP marker data was also used for estimating the genetic structure of the landraces using the Bayesian clustering model implemented in the computer software STRUCTURE, and logarithm probability relative to standard deviation (ΔK) was used to estimate the optimal value of cluster (K). Population structure of durum wheat landraces from Central Fertile Crescent was explained at different K values ranging from 3 to 5 (Fig 4). Most of the landraces (60%) were assigned to mixed groups (Cluster 3) at K = 3, K = 4 and K = 5, whereas rest of the 40% landraces was assigned to Cluster 1 and 2 at each K having a probability value higher than 80%. The percentage of assigned groups increased with the increase in value of K. Similarly, 4 subpopulations at K = 4 and five at K = 5 were observed in the structure analysis.

Average genetic distance among Turkish landraces was 0.516 and ranged from 0.029 to 0.698, whereas in case of Syria, it varied between 0.038 and 0.594. Genetic distance between both countries ranged from 0.025 to 0.680.

### Association among DArTseq, SNP markers and eco-geographical factors

**Fig 5:**
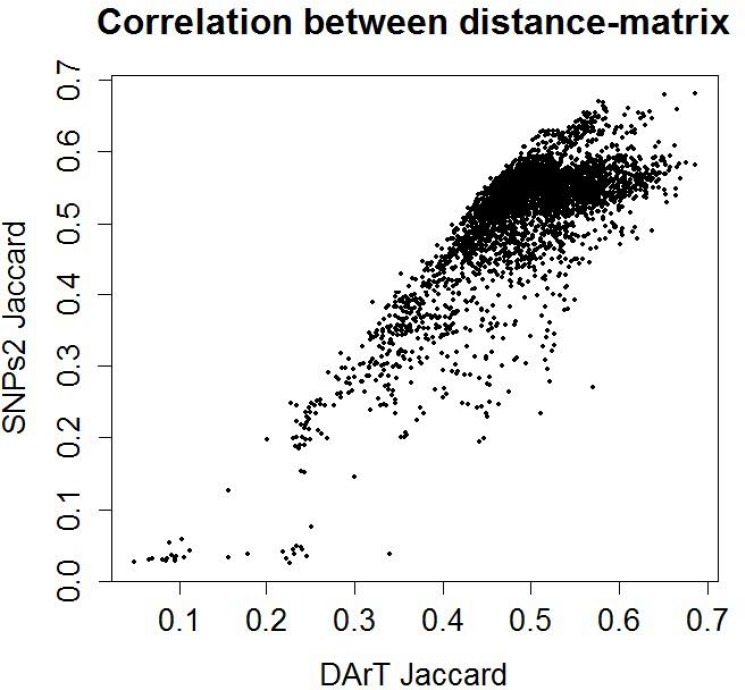
Mantel correlation test between DArTseq and SNP markers.

**Fig 6:**
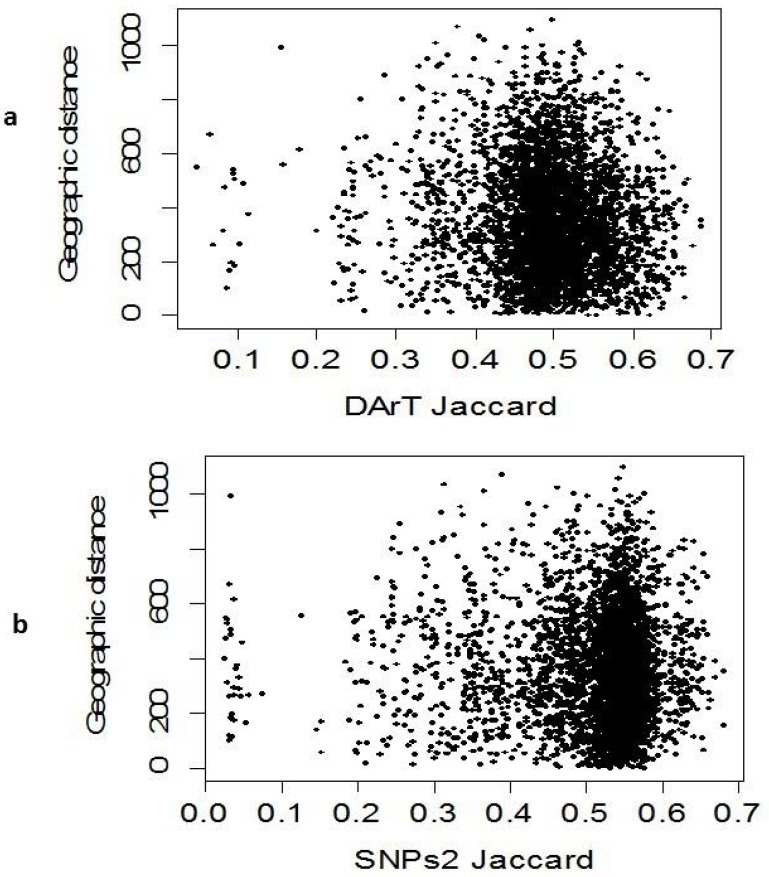
Association between geographical distance and genetic diversity based on (a) DArTseq and (b) SNP markers.

Comparisons among the clusters derived from DArTseq and SNP markers depicted high association between both markers system (r = 0.775; P < 0.001; Fig 5) through Mantel test showing a good fit between DArTseq and SNP marker data sets. The Mantel test was also conducted to find a correlation between genetic diversity and geographical coordinates. The results showed a non-significant correlation between geographical coordinates and DArTseq (r = -0.085) and SNP (r = -0.039) loci (Fig 6A and 6B).

## Discussion

Farmers have been growing the wheat landraces composed of traditional varieties through years of natural and human selection that are as a consequence adapted to local ecological conditions and management practices [24]. Earlier researchers [25, 26] demonstrated that the wild emmer domesticated in the Fertile Crescent and the subsequence breeding of domesticated durum and bread wheat led to the narrowing of their genetic diversity. It was evaluated that the initial loss in the diversity was 84% in durum wheat during domestication. Throughout their evolutionary history, wheat crops have been molded through the major contribution of farmers to meet the end user requirements, cultural practices, and to respond to growing conditions and changing socioeconomics [24].

Information on available genetic resources, their geographical locations, and understanding of their relationships can be used to gain insight into population divergence. Comprehensive knowledge about genetic diversity of durum wheat from various eco-geographical regions is expected to have a remarkable impact on the maintenance and usage of durum germplasm, facilitating breeders in devising approaches to achieve profitable diversification in the breeding programs. We tried here to give a brief summary of genetic diversity of selected 91 durum wheat landraces collected from its mega center of the diversity, Turkey and Syria. These landraces were collected from almost all durum wheat producing areas of the both countries.

Different molecular markers were used for assessment of genetic diversity in durum wheat such as AFLP, ISSR, RAPD, SSR etc. However, with the development of high-throughput methods it was made possible to genotype thousands of markers in single assay for many individual plants. The quick progress in sequencing and genotyping technologies in the last decade has permitted the advancement of SNP and DArTseq arrays even for polyploid crops such as wheat [27]. These new markers now rapidly started to be used in wheat and are accelerating the genomic research in many other crops.

Diversity Arrays Technology (DArTseq) and SNP have proved to be a robust molecular markers used for analysis of genetic diversity and genetic mapping in many crops [15,19,20]. The tree created using the Neighbor Joining clustering method on both sets of markers has shown the grouping of landraces into 3 major groups. (Fig 2A and 2B). Grouping of the landraces was almost the same with both types of markers system. However, grouping didn't show a relationship with their geographical origin. Similarly, the results of principal coordinate analysis (PCoA) were consistent with the Neighbor Joining analysis (Fig 3A and 3B).

DArTseq and SNP markers have not separated Turkish and Syrian durum wheat landraces according to their provenance and did not group them corresponding to their geographical origin. Landraces from Turkey and Syria were mixed in different clusters. In case of SNP analysis, all groups have shown patterns of clustering. Blend of the Turkish and Syrian landraces also explains nearby relationship of the durum wheat genetic resources from both countries. This close relationship among the durum wheat genetic resources of two countries is not surprising, as we clearly know that Karacadağ mountains in south eastern part of Turkey [5] and Northern part of Syria are the main centers of diversity and place of origin of wheat. This was based on the proposition that wild einkorn and wild emmer from this area are genetically more closely related to the domesticated crop plants than elsewhere. Based on that information, it can be concluded that this area is the center of diversity and domestication of wheat. Domestication and diversification are complex evolutionary processes in which the genetic forces of mutation, selection, migration and genetic drift have momentous role. Durum landraces were cultivated in these areas for thousands years, but there was no proper breeding program until few years ago. The seeds of durum wheat landraces were collected from the natural and mountainous areas and cultivated by ancient farmers. The breeding of commercial varieties just started after the input of CIMMYT and later establishment of ICARDA in the Syria. ICARDA uses genetic resources from this area and developed wheat cultivars for most of the developing world. The local farmers of these regions sow durum wheat each year and keep some of the seeds for the next season after harvesting. Moreover, Turkey and Syria were parts of the same country in near history. Possibly the indirect selection by the farmers with better adaptation to local agro-climatic conditions and exchange of seeds by farmers from distant regions could be the causes of this mixing of the landraces among both countries. Therefore, we can conclude that the dispersal and exchange of seeds followed by mixing was common among the farmers of Turkey and Syria. Transfer of landraces among regions, resulted by mixing and introgression with previous germplasm, could be an addition to this logic. Archaeobotany gives clues about humans imposing different and probably dynamic selective pressures on the plants being utilized. These activities occurred concurrently throughout a large region of the Fertile Crescent, with the resulting evolutionary trajectories possibly coming together in a complex manner.

The informativeness of the DArTseq and SNP markers was estimated by the Polymorphism information content (PIC). PIC provides the information about the diversity of a gene or DNA segment in a population which is used for indicating the evolutionary pressure on the allele and the mutations in a locus that might have happened over a time period. PIC value is calculated as a maximum of 0.5 when a marker is scored as 50% of 0 and 50% of 1. Average PIC values of the all DArTseq and SNP markers were 0.265 and 0.302 respectively. Overall, the distribution of PIC values was asymmetrical and skewed towards the lower values in both markers systems. We were not able to compare PIC values as there are only few reports on genetic diversity using DArT and SNPs and we could not trace any report using DArTseq markers in genetic diversity studies in durum wheat. Average PIC values obtained in our study by both markers systems were higher than the PIC value obtained by Ren et al. [2] using SNP markers, who studied world-wide durum collection of 150 genotypes and average PIC value was 0.18888. Moragues et al [28] studied the genetic diversity of 63 durum wheat landraces from the Mediterranean countries using AFLP and SSR markers, and average PIC values were 0.24 and 0.70 for AFLP and microsatellites, respectively. Distribution of PIC values among DArTseq and SNP markers is shown in the histogram (Fig 1A and 1B) Distribution of the PIC values among markers was almost the same and more than 3000 SNP and DArTseq markers had PIC values of 0.5. Most of the DArTseq and SNP markers have PIC values greater than 0.2.

In addition to the PIC value, some other quality parameters, such as call rate and reproducibility of the each marker within the panel of diverse landraces were also estimated. Call rate is the percentage of valid scores in all possible scores for a marker, where reproducibility is the measured in percentage of reproducibility of the scoring for replicated samples.

We described here a whole genome analysis of population structure among single selected plants from each durum wheat landrace representing the diversity of Turkey and Syria. The grouping of the landraces based on the Bayesian model showed the same results as reflected in Neighbor Joining analysis and Principal coordinate analysis. Groups A and B of cluster analysis (Figs 2A and 3B) could be detected in K = 4 and K = 5 groups in the STRUCTURE analysis. Cluster C further divided into C1 group that is mostly homogenous in nature, while the rest of the landraces in C cluster are heterogeneous in nature (Fig 4). The results of population structure analysis revealed that cluster 3 has the highest number of landraces and this cluster is a mixture of the different landraces. With increasing the number of K to 4 and 5, mixing of the landraces also increased (Fig 4). It is also interesting to note that STRUCTURE analysis allocated durum wheat landraces to same groups with both DArTseq and SNP markers dataset. This was confirmed by the Mantel test that was conducted to check association between both markers systems. Both marker platforms were developed through a type of genotyping by sequencing (GBS) technology named DArT sequencing, and provide high marker densities (tens of thousands of markers). The technology is hence established in the field of immense resolution mapping and comprehensive genetic dissection of traits.

In order to see whether or not genetic distance or genetic diversity is correlated with eco-geographical coordinates, we have conducted mental test to have a clear picture about the pattern of variations among landraces (Fig 6A and 6B). It was clear that no association was revealed based on Jaccard genetic distances and geographical coordinates (altitudes and latitudes), unlike the experience of Ren et al. 2013 [2], where it was observed that ecological factors along with geography, temperature, and water-availability factors, singly or in combination, explained a momentous portion of variation in SNP allele frequency in wild emmer depicting a broad range of environmental conditions in Israel and Turkey. Mixing of the Syrian and Turkish landraces is not surprising because about hundred years before, all of these areas were under the control of Ottoman Empire, and there was no particular breeding program adapted to local consumer needs. Old farmers were growing wheat from seeds obtained in the previous year harvest, while exchange of seeds among farmers was a frequent practice.

## Conclusion

This is the first report of a DArTseq analysis results for durum wheat landraces from the Central Fertile Crescent. The results illustrated here present an advantageous starting point for future genomics studies in durum wheat for trait of interest. Genomic selection has an advanced breeding tool that holds great potential for plant breeding, but which depends on a large number of molecular markers and having a relatively well-balanced genome-wide coverage [29]. The rapid advancement in sequencing and genotyping technologies in the last few years has enabled the development of SNP and DArTseq arrays even for polyploid crops such as wheat [27].

One of the most promising approaches for trait improvement is the introduction of novel alleles. As a first step, allele mining approaches can be performed in various ways and in different germplasm collections. Therefore, we are conducting country-wide multi-location field experiments with diverse environments for phenotyping the germplasm and are trying to identify the linked markers for genetic dissection of complex traits. In addition, genetic relationships could be used for the selection of parents for inclusion in the breeding program, if no pedigree data available. Here we have randomly selected single plants from each landrace and have provided the genetic diversity in a panel of landraces collected from the area, known as the most genetically diversified area or center of domestication. The information generated here could be used to design breeding programs adapted to local needs. The huge number of available DArTseq and SNP markers, their cost-effectiveness and relatively high polymorphism content are excellent aspects for extensive genome-wide screening for genetic diversity purposes. The results obtained with the DArTseq markers were in good agreement with those obtained with SNP, which might be due the possibility of genotyping 1000s of loci without any previous sequence information. The genetic diversity of the durum wheat gene pool must be further elucidated to facilitate classification, proper maintenance, conservation and utilization of these valuable resources.

## Supporting Information

S1 Table Raw sequence data of the DArTseq and SNP markers used for generating the marker data for 91 accessions. (XLSX)
